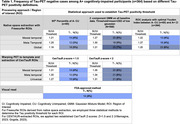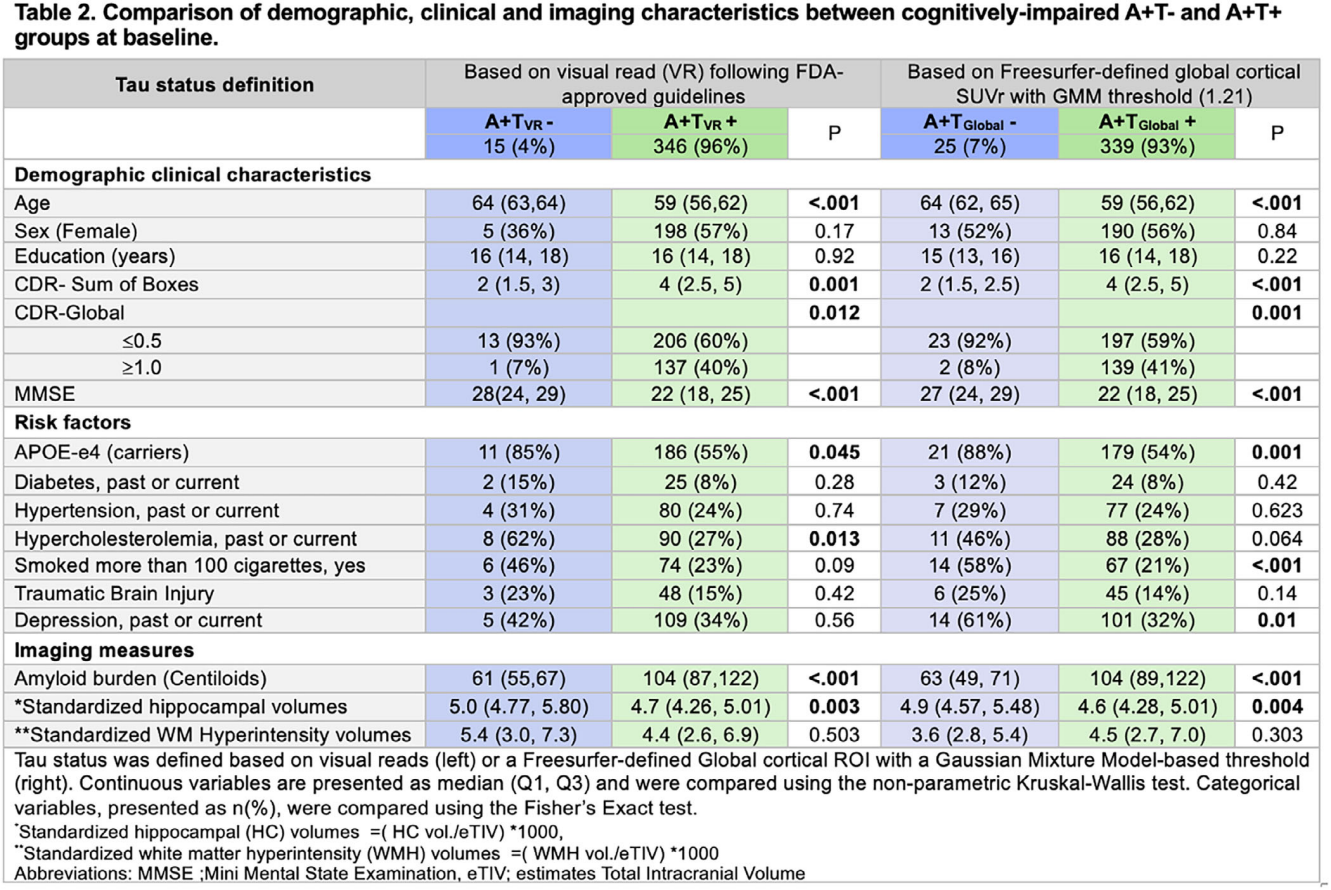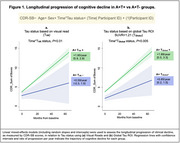# Characterizing patients with early onset Alzheimer's Disease and amyloid‐positive, tau‐negative (A+T‐) PET profiles: data from the longitudinal Early‐Onset Alzheimer's Disease Study (LEADS)

**DOI:** 10.1002/alz70856_103476

**Published:** 2025-12-26

**Authors:** Maison Abu Raya, David N. Soleimani‐Meigooni, Ganna Blazhenets, Konstantinos Chiotis, Julien Lagarde, Agathe Vrillon, Piyush Maiti, Charles C. Windon, Ehud Zeltzer, Jiaxiuxiu Zhang, Claire Yballa, Ranjani Shankar, Alinda Amuiri, Salma Rocha, Dustin B. Hammers, Ani Eloyan, Robert A. Koeppe, Maria C. Carrillo, Brad C. Dickerson, Liana G. Apostolova, Gil D. Rabinovici, Renaud La Joie

**Affiliations:** ^1^ Global Brain Health institute‐ UCSF, San Francisco, CA, USA; ^2^ University of california San Francisco, San Francisco, CA, USA; ^3^ UCSF Alzheimer's Disease Research Center, San Francisco, CA, USA; ^4^ Memory and Aging Center, Weill Institute for Neurosciences, University of California San Francisco, San Francisco, CA, USA; ^5^ Lawrence Berkeley National Laboratory, Berkeley, CA, USA; ^6^ Neurology of Memory and Language Department, GHU Paris Psychiatrie & Neurosciences, Hôpital Sainte‐Anne, Paris, France; ^7^ Global Brain Health Institute, San Francisco, CA, USA; ^8^ Memory and Aging center, UCSF, San Francisco, CA, USA; ^9^ Department of Neurology, University of California, San Francisco, San Francisco, CA, USA; ^10^ Global Brain Health Institute, San francisco, CA, USA; ^11^ University of California, San Francisco, San Francisco, CA, USA; ^12^ Memory and Aging Center, Weill Institute for Neurosciences, University of California, San Francisco, San Francisco, CA, USA; ^13^ Indiana University School of Medicine, Indianapolis, IN, USA; ^14^ Department of Biostatistics, Brown University, Providence, RI, USA; ^15^ University of Michigan, Ann Arbor, MI, USA; ^16^ Alzheimer's Association, Chicago, IL, USA; ^17^ Massachusetts General Hospital and Harvard Medical School, Boston, MA, USA; ^18^ Department of Neurology, Indiana University School of Medicine, Indianapolis, IN, USA; ^19^ Department of Radiology and Biomedical Imaging, University of California San Francisco, San Francisco, CA, USA; ^20^ Memory and Aging Center, UCSF Weill Institute for Neurosciences, University of California, San Francisco, San Francisco, CA, USA; ^21^ Global Brain Health Institute, UCSF, San Francisco, CA, USA

## Abstract

**Background:**

The interpretation of an A+T‐ PET profile in clinically impaired individuals can make it difficult to identify underlying etiology and predict future outcomes. We aimed to assess the frequency of A+T‐ profiles within a cohort of patients diagnosed with early‐onset Alzheimer's Disease and describe their clinical and imaging characteristics.

**Method:**

We analyzed 568 LEADS participants with baseline [^18^F]florbetaben and [^18^F]flortaucipir PET: 483 cognitively‐impaired (CI, i.e., clinical diagnosis of MCI or dementia) and 85 cognitively unimpaired (CU) participants. Amyloid positivity (A+) was based on [^18^F]florbetaben‐PET quantification using a 25 Centiloid threshold. To test the robustness of our findings, tau‐PET status was determined in several complementary ways. First, based on an expert visual read following FDA‐approved guidelines. Second, based on Flortaucipir‐SUVR extracted from various regions of interest (in native space using Freesurfer or in template space with CenTauR procedures) and thresholded using different statistical methods (Table 1). We then compared baseline characteristics between the A+T‐ and A+T+ groups using Kruskall‐Wallis or Fisher exact tests and assessed differences in longitudinal clinical decline using linear mixed effect models (outcome: CDR‐SB).

**Result:**

Among 364 A+ CI participants, rates of negative tau‐PET were minimal (3‐7%), regardless of the tau‐PET approach (Table 1). Visual reads were highly concordant with tau status defined by quantitative approaches (agreement=96‐99%, Kappa=0.48‐0.84). Comparison between the A+T+ and A+T‐ groups was performed twice, based on the two methods that provided the divergent T‐ rates: visual reads (4%) or global cortical SUVR with a Gaussian Mixture Model‐based threshold (7%). Regardless of the tau status definitions, the A+T‐ group exhibited milder clinical impairment than the A+T+ group despite being older and having a higher frequency of APOE‐e4, hypercholesterolemia, and tobacco smoking (Table 2). The A+T‐ group had lower amyloid burden and larger hippocampal volumes than A+T+ patients but similar white matter hyperintensity burden. Longitudinally, the A+T‐ group **showed a significant increase in CDR‐SB, but clinical progression was slower than the A+T+** group (Figure 1).

**Conclusion:**

In amyloid‐positive patients with EOAD, tau‐PET results were predominantly positive regardless of tau‐status definitions (93‐97%). The rare A+T‐ profile may reflect early disease stages, with tau pathology below PET detection levels.